# Changes in Gut Microbiota by the *Lactobacillus casei* Anchoring the K88 Fimbrial Protein Prevented Newborn Piglets From Clinical Diarrhea

**DOI:** 10.3389/fcimb.2022.842007

**Published:** 2022-03-18

**Authors:** Da Qin, Yongfei Bai, Yan Li, Yanmei Huang, Liyang Li, Guihua Wang, Yi Qu, Jiabin Wang, Li-Yun Yu, Xilin Hou

**Affiliations:** ^1^ College of Life Science and Technology, Heilongjiang Bayi Agricultural University, Daqing, China; ^2^ Colleges of Animal Science and Technology, Heilongjiang Bayi Agricultural University, Daqing, China

**Keywords:** r*Lactobacillus casei*, ETEC K88, 16S rRNA sequencing, newborn piglets, gut microbiota, diarrhea

## Abstract

In the last 20 years, accumulating evidence indicates that the gut microbiota contribute to the development, maturation, and regulation of the host immune system and mediate host anti-pathogen defenses. *Lactobacillus casei* (*L.casei*) is a normal flora of the gastrointestinal tract in mammals and, as a great mucosal delivery vehicle, has wide use in bioengineering. However, the diarrhea prevention role of commensal intestinal microbiota interfered by the recombinant *L.casei* (r*L.casei*) in newborn piglets is not well understood. In our study, newborn piglets orally fed with the r*L.casei* surface displayed the fimbrial protein K88 of enterotoxigenic *Escherichia coli* (ETEC) and their feces were collected for a period of time after feeding. The next-generation sequencing of these fecal samples showed that the relative abundance of *L.casei* was significantly increased. The oral administration of r*L.casei* altered the intestinal microbial community as evidenced by altered microbial diversity and microbial taxonomic composition. Remarkably, the functional enhancing of the intestinal bacterial community by r*L.casei* was positively correlated with membrane transport, replication, and repair (p < 0.05). The specific antibody detection indicates that high levels of anti-K88 secretory immunoglobulin A (sIgA) were induced in fecal samples and systemic immunoglobulin G was produced in serum. The diarrhea rate in piglets caused by ETEC K88 was decreased by about 24%. Thus, the oral administration of r*L.casei* not only activated the mucosal and humoral immune responses *in vivo* but also contributed to shape the intestinal probiotics in newborn piglets and to significantly reduce the diarrhea rates of newborn piglets.

## 1 Introduction

Enterotoxigenic *Escherichia coli* (ETEC), one of the main pathogenic bacteria, can cause fatal diarrhea and edema in neonatal and weaning piglets, which leads to high morbidity and mortality around the world, especially in developing countries (Moon et al., 1979; Dorsey et al., 2010; Ikwap et al., 2016). The pathogens ETEC are non-invasive bacteria that colonize the small intestine *via* pili or fimbriae, where they produce enterotoxin leading to severe diarrhea that is fatal to piglets. The pili adhesins known to be important in ETEC infection to neonatal animals are K88 (F4), K99 (F5), 987P (F6), and F41 (F7) which are the key antigens inducing neutralizing antibodies (Moon et al., 1977; Morris et al., 1983; Sahagun-Ruiz et al., 2015).

It has been reported that the intestinal microbiota could resist the invasion of pathogenic microorganisms and assist the host immune system to eliminate exogenous pathogenic microorganisms (De Filippo et al., 2010; Wu et al., 2011; Gresse et al., 2017). All of the host’s diet, lifestyle, external environment, and genetic susceptibility affect the composition of the intestinal microbiota (Chen et al., 2018; Li et al., 2018). However, over time, the host intestinal microbiota remained stable (Guevarra et al., 2019). *Lactobacillus casei*, a normal resident of the gastrointestinal tract of mammals, has been extensively studied over the past few decades for its probiotic properties in clinical and animal models (McFarland et al., 2018; Riaz Rajoka et al., 2018). Also, it may be a good choice for mucosal immunization (Friedman et al., 2000; Lee et al., 2000; Shata and Hone, 2001; Wang et al., 2016), because it is safe, cheap, stable, and easily administered and exhibits adjuvant properties (Pouwels et al., 1998; Seegers, 2002; Praveen et al., 2004; Tarahomjoo, 2012). The potentiality of *L.casei* to deliver heterologous antigens to the mucosal immune system has been investigated during the last decades (Pouwels et al., 1996; Ho et al., 2005; Kuczkowska et al., 2017; LeCureux and Dean, 2018). Previously, for surface display of the antigens ETEC K88 and K99 on *L.casei*, we have developed the strategy of generating *Lactobacillus casei*/*E. coli* shuttle expression vector with the PgsA gene as an anchoring matrix (Wen et al., 2012). However, we do not know whether this live recombinant *L.casei* affects the intestinal flora of animals, especially piglets. In this study, we mainly investigated the changes of immunoglobulin and intestinal flora after orally immunizing to piglets the recombinant pLA-ETEC K88/*L.casei*. The bioinformatics analysis may clear in focus the potential for utilizing this recombinant *L.casei* to interfere the intestinal physiological characteristics in newborn piglets and can reveal the specific effects on the intestinal flora.

## 2 Materials and Methods

### 2.1 Bacterial Strains and Growth Conditions

Recombinant strain pLA-ETEC K88/*L.casei* was constructed and stored in our laboratory (Wen et al., 2012). Briefly, The 851-bp DNA fragment encoding the fimbrial protein K88 (GenBank: M29375.1) was amplified from the ETEC strain by PCR, and then the PCR product was inserted into the vector pLA to construct the recombinant plasmid pLA-K88. Electroporation of *Lactobacillus casei* (ATCC-334) was carried out according to the transformation condition which was 2.0 kV/cm, 200 Ω, 25 μF, by using a Gene Pulser (Bio-Rad, Richmond, CA). It grew anaerobically at 37°C with 34 mg/ml of chloromycetin (Cm; Sigma) in MRS broth medium (Difco).

### 2.2 Animals, Diets, and Sampling

A total of 48 newborn piglets from 5 litters (Landrace, newborn average litter weight, 1.90 ± 0.05 kg) were obtained from the Sanhe farm (Qiqihar, Heilongjiang Province, China). All pigs in this study were chosen from one delivery room and had similar genetic backgrounds and husbandry practices. These piglets were allocated randomly to two groups (Ctrl: no feeding pLA-ETEC K88/*L.casei*, OA: feeding pLA-ETEC K88/*L.casei* on days 1–5) for the 28-day experiment. The piglets in the two groups were placed in three pens with a similar environment, the room temperature was maintained at 30°C, and the humidity was maintained constant at 65%–75%. The 24 piglets in the Ctrl group were fed the basic diet without any probiotics in the 28-day experiment. For the treatment group, 24 piglets in the OA group were treated with the protocol described by Li-Juan Wen (Wen et al., 2012). Briefly, pLA-ETEC K88/*L.casei* (5 × 10^11^ CFU/ml) cells were orally administered daily on days 0–5 and treated according to animal protocols approved by the Institutional Animal Care and Use Committee (IACUC). All of the newborn piglets used in this study were weighed at birth, days 15 and 28 of age. The weights were calculated according to groups ctrl and OA no matter with or without diarrhea.

For the next-generation sequencing, five piglet samples were chosen randomly in each group. Piglets’ fecal samples (200 mg) were collected in a cryopreservation tube (Axygen) on days 15 and 28 for different groups. The Ctrl group samples and OA group samples were both collected on days 15 and 28 and named Ctrl15, Ctrl28, OA15, and OA28. After sample collection, the samples were quickly placed into the sterile tubes, then thrown into liquid nitrogen for half an hour and stored at -80°C until sequencing.

For special antibody detection, the piglets’ fecal samples (200 mg) were collected in the cryopreservation tube (Axygen) on days 0 (preimmune), 5, 10, 15, 20, and 28 for the Ctrl group and OA group. After collection, the samples were quickly placed into the sterile tubes, then thrown into liquid nitrogen for half an hour and stored at -80°C until antibody detection. The blood samples were collected from the front cavity vein of piglets on days 0 (preimmune), 5, 10, 15, 20, and 28 for the Ctrl group and OA group. Serum was isolated from the blood and stored at -20° until analysis.

The incidence of diarrhea was recorded twice a day (monitoring time: 9:00 a.m. and 4:00 p.m.) according to the method of Ou et al. (Ou et al., 2007). In detail, scores were assessed as 0 = normal, solid feces; 1 = slight diarrhea, soft and loose feces; 2 = moderate diarrhea, semi-liquid feces; or 3 = severe diarrhea, liquid and unformed feces. Diarrhea was defined as a score of 2 or 3 per day, and the incidence of diarrhea (%) was calculated as (number of piglets with diarrhea)/(total number of experiment piglets) × 100%. On days 0 (preimmune) and 28, the piglets were weighed.

According to the score of piglet diarrhea, we found that six piglets had obvious symptoms of diarrhea, and then piglet feces were collected.

### 2.3 PCR

In order to determine the diarrhea symptoms in the newborn piglets caused by ETEC K88, we conducted PCR-specific detection of feces collected from diarrheal piglets. We firstly extracted DNA from the feces samples, then used the designed K88 gene primers (5′-CGCGGATCCTTT GGTAATGTATTGAATG-3T, 5′-CGGGGTACCTTACTCTTTGAATCTGTC-3′) to amplify by PCR. The PCR products were detected by 1% agarose gel.

### 2.4 ELISA

The specific antibodies IgG, IgA in serum, and sIgA in fecal samples were determined by enzyme-linked immunosorbent assay (ELISA), as described previously (Bai et al., 2020). The optical density was measured at 450 nm by using an ELISA auto-reader (Molecular Devices, San Jose, CA, USA) and visualized by R software (version 3.6.1) with ggplot2 package.

### 2.5 Next-Generation Sequencing of the 16S rRNA Gene

Genomic DNA was extracted from each group at each time point. The 16S rRNA genes were amplified *via* a pair of the universal bacterial 16S primers 338F (5′-ACTCCTACGGGAGGCAG CAG-3′) and 806R (5′-GGACTACHVGGGTWTC TAAT-3′), covering the V3–V4 regions of the 16S rRNA gene. The sequencing library was quantified by Qubit and qPCR, and the barcode V3 and V4 PCR amplicons were sequenced using the Illumina MiSeq platform (Shanghai Personal Biotechnology Co., Ltd., Shanghai, China). All the raw sequencing data were submitted to the NCBI Sequence Read Archive (SRA) database under accession nos. SRP282258 and SRP344853.

### 2.6 Sequence Processing and Bioinformatics Analysis

#### 2.6.1 Taxonomy classification

The sequences used in the subsequent analysis (effective tags) were obtained by successively splicing raw sequence reads using FLASH software (version 1.2.7) (Lozupone and Knight, 2005). After quality filtering by the QIIME quality-control process (Caporaso et al., 2010), the repetitive sequences were removed from the effective tags to acquire representative sequences by using USEARCH software (version v5.2.236) (Caporaso et al., 2010). The representative sequences showing 97% identity were then clustered as operational taxonomic units (OTUs) using UCLUST (Edgar, 2010). The taxonomy of each 16S rRNA gene sequence was analyzed by RDP Classifier against the SILVA (Release 115) 16S rRNA gene database (Quast et al., 2013). OTUs with abundance values less than 0.001% of the total sequencing samples were removed (Bokulich et al., 2013), and this abundance matrix with rare OTUs removed was used for subsequent series of analyses.

#### 2.6.2 Alpha and Beta Diversity Analysis

Alpha diversity can be used to measure the diversity and abundance of microbes. Various alpha diversity indexes, such as Chao1, Shannon, Simpson, and ACE, were calculated on the basis of QIIME software (version 1.7.0), and boxplots were drawn *via* R software (version 3.6.1) with the ggplot2 package. To compare the construction of the microorganisms between the different samples, weighted UniFrac distances were calculated by using QIIME (version 1.7.0) (Lundberg et al., 2013). In addition, the samples were clustered basing on the weighted UniFrac distance matrix using the principal coordinate analysis (PCoA) (Ramette, 2007) and non-metric multidimensional scaling (NMDS), then visualized by R software (version 3.6.1) with the ggplot2 package. Also, the samples could be clustered based on both the UniFrac distance matrix using the unweighted pair-group method with arithmetic mean (UPGMA) implemented in QIIME (version 1.7.0) (Lozupone and Knight, 2005; Geng et al., 2018).

#### 2.6.3 Random Forest Regression Analysis

In order to screen the bacterial genera that contribute most to the impact of intestinal flora, we regressed the relative abundances of bacterial taxa for all groups using the R algorithm (Random Forest) in default parameters which are described by Jingying Zhang (Zhang et al., 2018). Random forests are a classical and efficient machine learning algorithm based on the decision tree, which belongs to the non-linear classifier and can deeply mine the complex non-linear interdependence among variables, especially for microbiota data that often present discrete and discontinuous distribution (Breiman, 2001; Wang et al., 2018b). We used the “Random Forest” package in R software to build a Random Forest classification list by characteristic importance which was determined in 100 iterations. The number of marker taxa was identified using 10-fold cross-validation implemented with the “rfcv ()” function in the R package “Random Forest” with five repeats (Breiman, 2001; Zhang et al., 2018). Then the mean decrease in accuracy (the contribution of each genus to the model prediction accuracy) of every genus was visualized by R software (version 3.6.1) with the ggplot2 package.

#### 2.6.4 Microbial Function Prediction

To find out the potential functional profiles of the bacterial community, the sequences were clustered into OTUs at 97% similarity by using a closed-reference approach in the Greengenes 13.5 database *via* QIIME (version 1.7.0). The resulting OTUs were used for the prediction of microbial function in PICRUST (Langille et al., 2013) according to the online protocol. Briefly, after the OTUs were normalized by 16S rRNA gene copy number, the metagenome for each sample was predicted, and the accuracy of the metagenome predictions was assessed. The relative abundance of each KEGG level was visualized by R software (version 3.6.1) with the ggplot2 package. The differences in KEGG level between the control and experimental groups were assessed by STEMP software (Langille et al., 2013).

### 2.7 Statistical Analysis

The Student’s t-test was used to evaluate differences in fecal microbiome between the control and experimental groups, growth details, differences in the relative abundances of the genes involved in the KEGG pathway, and correlation analysis. Differences were considered significant when the *p* value was <0.05.

## 3 Results

### 3.1 Growth Performance, Systemic, and Mucosal Immune Responses Induced by Oral Immunization With the Recombinant *L.casei*

**Figure 1:**
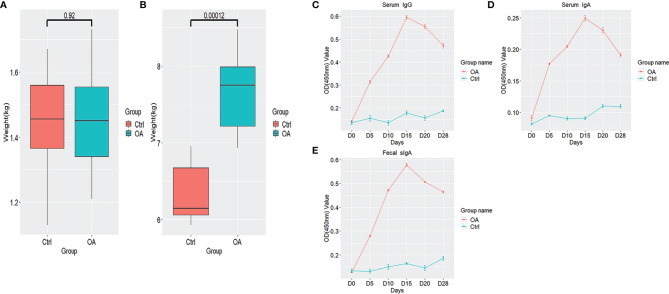
Piglets’ weight detail and fimbrial K88-specific antibody responses in serum and fecal samples. Piglets’ weight detail in days 0 **(A)** and piglets’ weight detail in days 28 **(B)** are shown by boxplots. Specific IgG antibody in serum samples **(C)**, specific IgA antibody in serum samples **(D)**, and specific sIgA antibody in fecal samples **(E)** based on optical density by ELISA. All diagrams were shown by R software (version 3.6.1) with the ggplot2 package. The boxplots represent the diversity measures for the 5 analysis groups (center line, median; box limits, first and third quartiles; whiskers, 1.5 × interquartile range). All outliers are plotted as individual points. The number up to the two groups means p value which is calculated by the Student’s t-test.

The 48 newborn piglets were grouped as Ctrl group (24 piglets) and OA group (24 piglets). The piglets in the Ctrl group were fed the normal diet without any probiotics and those in the OA group fed pLA-ETEC K88/*L.casei* at days 0–5. On day 0, there was no significant difference in average body weight of piglets between the Ctrl group and OA group (*p* > 0.05) (**Figure 1A**). However, there was a significant difference in average body weight between the Ctrl group and OA group on day 28 (*p* < 0.05) (**Figure 1B**). These results indicated that the recombinant *L.casei* can improve and promote the growth and development of newborn piglets.

The specific anti-K88 IgG antibody in serum samples from immunized piglets was determined (**Figure 1C**). After the oral immunization, the anti-K88-specific IgG level was higher in the OA group than that in the Ctrl group and the highest value was found on day 15.

In order to determine the mucosal immune response, the specific sIgA level in fecal samples and the IgA level in serum samples were measured by ELISA (**Figures 1D, E**). The results showed that the levels of the specific sIgA and IgA were all higher than the control, suggesting that the systemic and mucosal immune responses were induced by oral immunization with pLA-ETEC K88/*L.casei*.

### 3.2 Statistics of Diarrhea Rate and PCR Identification of Diarrhea Samples

**Figure 2:**
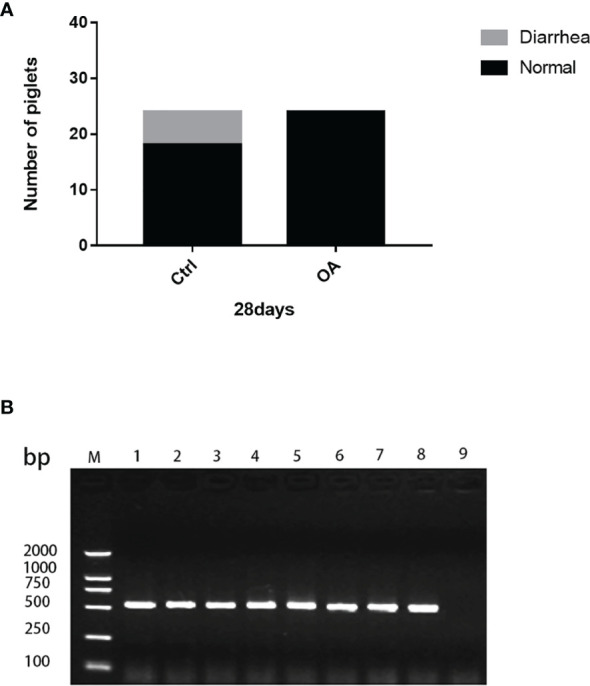
The diarrhea occurring rate in piglets **(A)**. PCR identification of diarrhea samples **(B)**. The diarrhea rate in the control group was 25%, but that in the treatment group did not occur **(A)**. Lanes 1–6, PCR templates from diarrhea piglet feces; Lane 7, r*L.casei* plasmid template as positive control; Lane 8, ETEC K88 as positive control; Lane 9, negative control. The ETEC K88 gene was amplified by PCR, suggesting that the symptoms of piglets’ diarrhea were caused by ETEC K88 **(B)**.

During the 28 days of the experiment, in the Ctrl group, there were 6 piglets that developed diarrhea (levels 2–3) caused by ETEC K88, which was confirmed by PCR detection (**Figure 2B**), and the diarrhea incidence was 25% (**Figure 2A**). However, in the OA group there was none. Thus, pLA-ETEC K88/*L.casei* improved the clinical performance of piglets by reducing incidence of diarrhea and morbidity.

### 3.3 α-Diversity and β-Diversity in Piglet Intestinal Flora

**Figure 3:**
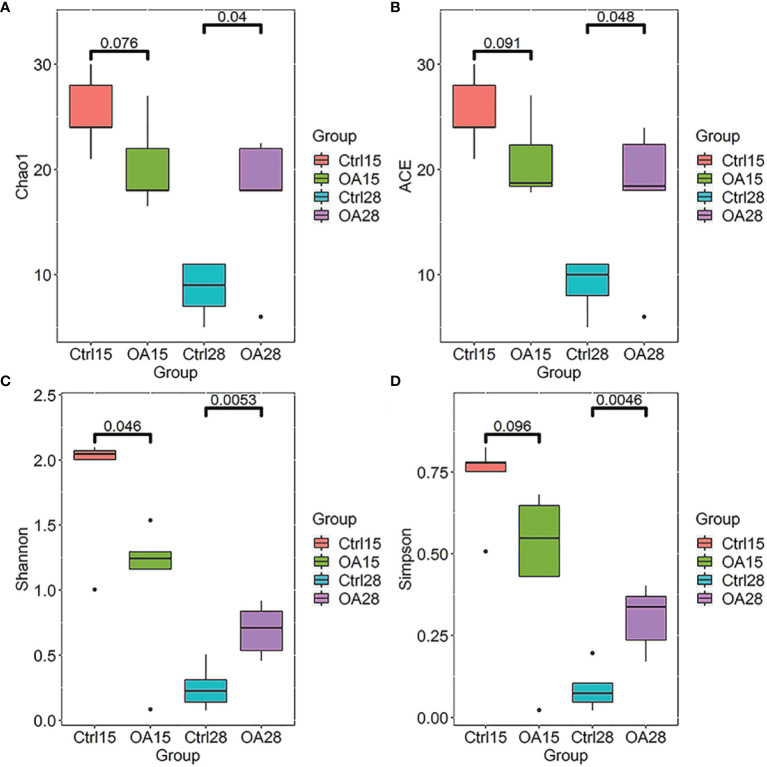
The α-diversity index of the intestinal flora in piglets. The Chao1 diversity **(A)**, ACE diversity **(B)**, Shannon diversity **(C)**, and Simpson diversity **(D)** based on OTU relative abundances were shown by R software (version 3.6.1) with the ggplot2 package. The boxplots represent the diversity measures for the 5 analysis groups (center line, median; box limits, first and third quartiles; whiskers, 1.5 × interquartile range). All outliers are plotted as individual points. The number up to the two groups means *p* value which is calculated by the Student’s t-test.

The intestinal flora in the three groups of piglets was analyzed by sequencing the bacterial 16S rRNA gene amplicons (V3+V4 region). After removing the low-quality sequences, 1,050,897 clean tags were identified as a total of 73,146 OTUs presenting in these samples. In α-diversity, the Chao1 and ACE indexes revealed the minor change but not statistical significance in the OA15 group when compared with the Ctrl15 group. Most interestingly, the Chao1 and ACE values of the α-diversity index were statistically significant in the OA28 group in comparison with the Ctrl28 group (*p* < 0.05). The Shannon and Simpson indexes of the α-diversity index were statistically significant in the OA28 group in comparison with the Ctrl28 group (*p*<0.05) (**Figure 3**). These data suggest that after the recombinant *L.casei* treatment, there was no clear difference in inter-group for the richness and variation of the intestinal flora on day 15. However, there was a significant difference in inter-group for the richness and variation of the intestinal flora on day 28.

**Figure 4:**
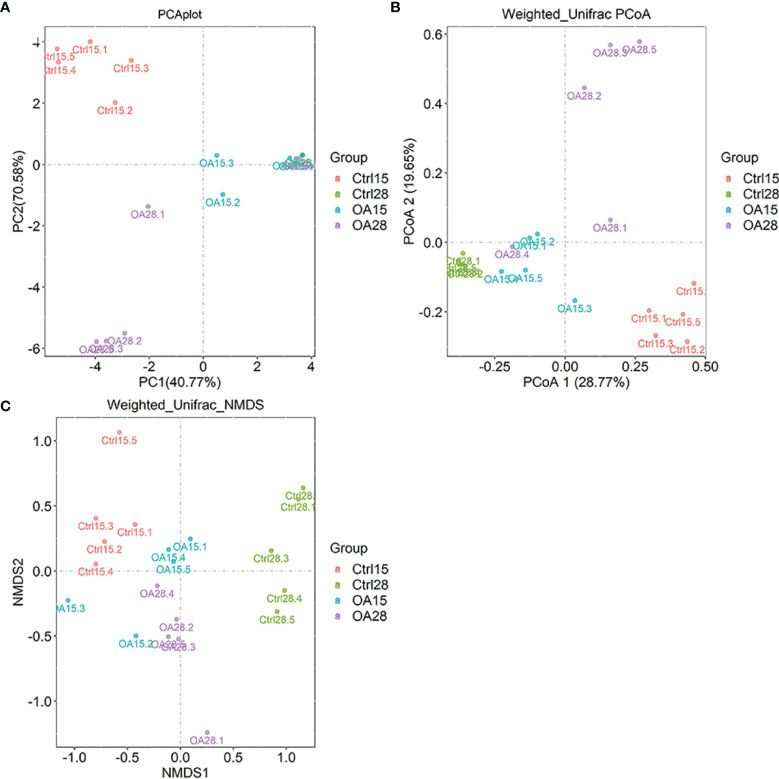
The β-diversity in piglet intestinal flora. PCA plot **(A)** was based on OTU relative abundances, and samples are colored according to each sample group. PCoA plot **(B)** and NMDS plot **(C)** based on weighted UniFrac distances between samples calculated using OTU relative abundances. The variance explained by each axis is stated as a percentage in parentheses.

In analyzing the degree of similarity between each group, principal component analysis (PCA) based on OTU relative abundances was taken for this experiment. In the coordinate system, the closer the two points, the higher the similarity was. The analysis results showed that the relative dispersion between different groups was relatively concentrated in the same group. According to the results of PCA analysis, there were significant differences between the Ctrl15 group and treatment group (OA group) (**Figure 4A**). Weighted UniFrac distances were used to estimate β–diversity and to compare among the three groups. The PCoA plot of the weighted UniFrac distances showed that the OA groups tended to separate from the Ctrl group (**Figure 4B**). Similar results were also observed in the analysis *via* non-metric multidimensional scaling (NMDS) (**Figure 4C**).

**Figure 5:**
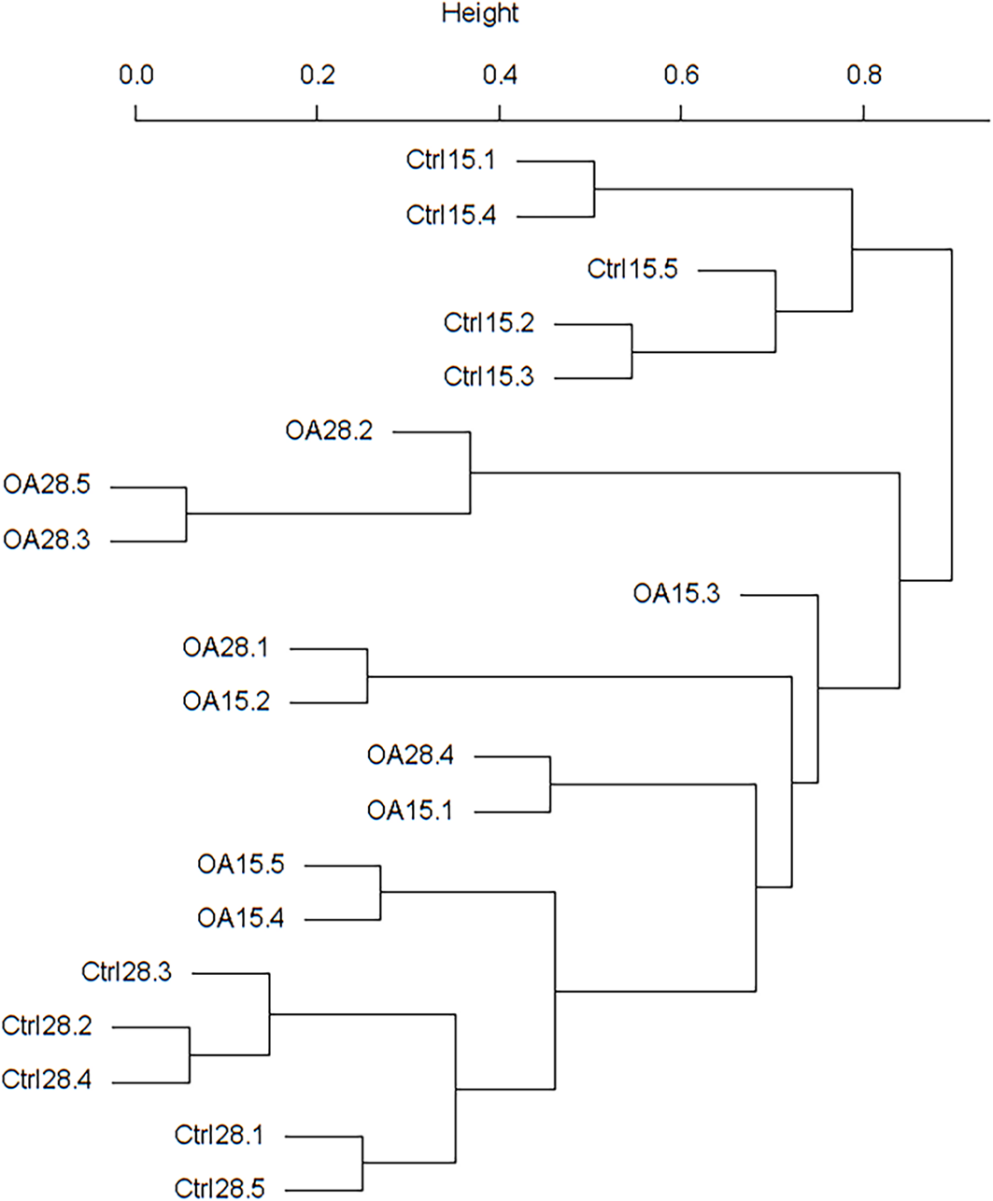
UPGMA phylogenetic tree constructed based on weighted UniFrac distances.

To determine the degree of similarity among the samples, a clustering tree of the samples was constructed (**Figure 5**). We found out that the treatment component was divided into two clusters, and the Ctrl15 component was divided into one cluster. However, three samples of the OA15 group were in the OA28 cluster. It was supposed that the intestinal microflora of piglets in the OA28 group was similar to that in the OA15 group.

### 3.4 Characterization of the Intestinal Flora of Piglets After the Recombinant *L.casei* Treatment

**Figure 6:**
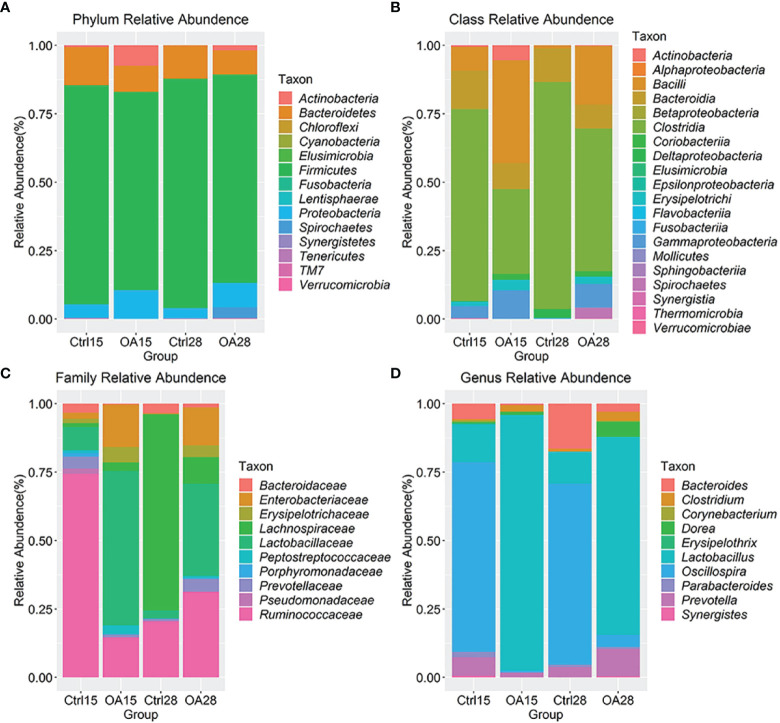
The intestinal flora detail of piglets after *L.casei* treatment, microbiota at the Phylum level **(A)**, Class level **(B)**, Family level**(C)** and Genus level **(D)** were shown by bar plot. The top 20 taxons of Family and Genus abundance were taken to analyse the effect of probiotic *L.casei* on intestinal flora composition in piglets.

We measured the relative abundance of intestinal flora in levels of phylum (**Figure 6A**), class (**Figure 6B**, family (**Figure 6C**), and genus (**Figure 6D**). The abundance of the two major phyla *Firmicutes* and *Proteobacteria* in the intestinal flora was 80.06% and 4.72% of the total abundance in the Ctlr15 group, respectively. However, after the recombinant *L.casei* treatment, the phyla *Firmicutes* was decreased in the OA15 group which constituted 72.26% and *Proteobacteria* was increased to 9.43%. Notably, in the OA28 group, the phyla *Proteobacteria* was decreased compared to the OA15 group, which constituted 8.88% of the total abundance. The abundance of the phyla *Firmicutes* was increased on day 28 (**Figure 6A**).

At the Class level (**Figure 6B**), the intestinal flora was dominated by class *Clostridia* which constituted 29.23% of the total abundance in the Ctlr15 group. After the recombinant *L.casei* treatment, the class *Bacilli* was significantly increased in the OA15 group which constituted 37.57% of their total abundance, and *Gammaproteobacteria* was dramatically increased to 10.29% in their total abundance.

At the Family level (**Figure 6C**), we took the top 10 taxons of total abundance. The intestinal flora was dominated by the families *Ruminococcaceae* and *Lactobacillaceae*, which constituted 65.75% and 7.56% of the total abundance in the Ctlr15 group. After feeding the recombinant *L.casei*, the family *Lactobacillaceae* was increased in the OA15 group which constituted 37.02% of their total abundance and the family *Ruminococcaceae* was dramatically decreased to 9.62% in their total abundance. However, surprisingly, *Lactobacillaceae* decreased to 20.27% in total abundance in the OA28 group.

At the Genus level (**Figure 6D**), we took the top 10 taxons of total abundance. After the recombinant *L.casei* treatment, *Lactobacillus* was markedly elevated in the OA15 group which achieved 37.02% of the total abundance. As unexpected, *Lactobacillus* decreased to 20.27% in total abundance in the OA28 group. Altogether, these results indicate that the recombinant *L.casei* intervention altered the microbiota composition in the piglet gut.

**Figure 7:**
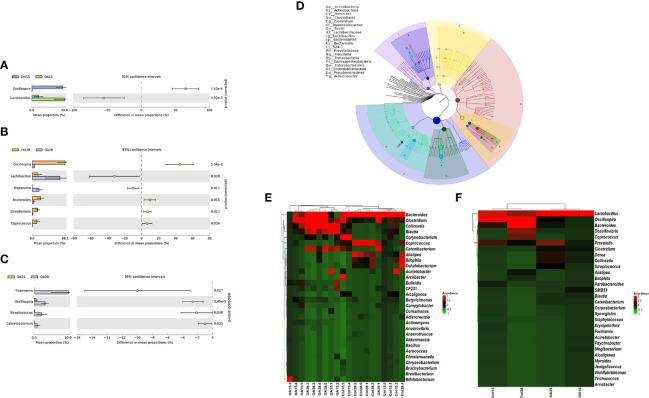
Based on the OTU relative abundance of each sample, the differences of bacterial abundances among the Ctrl15 group, Ctrl28 group, and the first feeding groups OA15 and OA28 were analyzed by using STAMP software with two-sided Student’s t-test. Ctrl15 group vs. OA15 group **(A)**, Ctrl28 group vs. OA28 group **(B)**, OA15 group vs. OA28 group **(C)**. GraPhlAn tools were used for visualization of the hierarchical tree **(D)**. The hierarchical tree shows the hierarchical relationship of all taxons (represented by nodes) from phylum to genus (arranged from inner circle to outer circle in turn), and the node size corresponds to the average relative abundance of the taxon. The top 20 taxons of relative abundance will also be identified by letters in the figure (arranged from phylum to genus in order from outer layer to inner layer). The top 30 taxons of genus abundance were taken to analyze the differences between each sample **(E)** and each group **(F)** by heat maps which were drawn by R software (version 3.6.1) with the ggplot2 package.

Basing on the OTU relative abundance of each sample, we analyzed the differences of bacterial abundances in genus level among groups Ctrl15, Ctrl28, OA15, and OA28 by using STAMP software with two-sided Student’s t-test (**Figures 7A, B**). The genus was ordered by effect sizes. In the Ctrl15 vs. OA15 group, the mean percentage of the genus *Lactobacillus* in the OA15 group was significantly higher than that in the Ctrl15 group (*p* < 0.05) (**Figure 7A**). In the Ctrl28 vs. OA28 group, the mean proportions of the genera *Lactobacillus* and *Treponema* in the OA28 group were upregulated in comparison to the Ctrl28 group (*p* < 0.05) (**Figure 7B**). However, it is not what we expected; the mean percentage values of the genus *Treponema*, *Oscillospira*, *Streptococcus*, and *Catenibacterium* in the OA28 group were upregulated when compared to those in the OA15 group (**Figure 7C**). This phenomenon also appeared to be consistent with the analysis results of the species classification tree (**Figure 7D**) and heat maps (**Figures 7E, F**) basing on OTU relative abundance between each sample.

**Figure 8:**
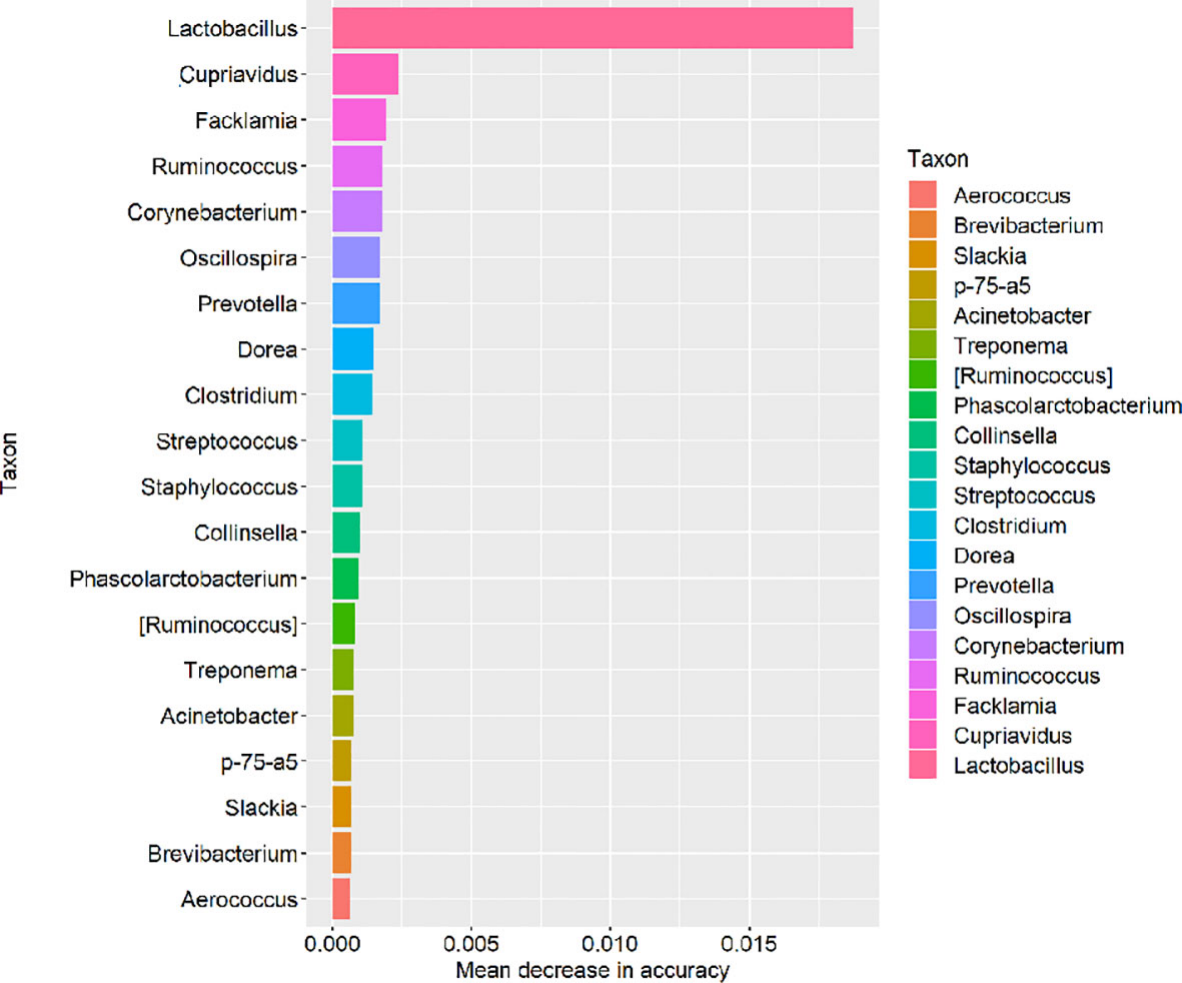
Based on the relative abundances of bacterial taxa, random forest regression analysis was taken and the top 20 taxons of genus in mean decrease in accuracy were visualized by R software.

The relative abundances of bacterial taxa and random forest regression analysis were carried out in each group to screen the key species affecting the change of intestinal flora. If a genus was removed, the model could predict the magnitude of the increase in error rate to determine its importance value which was ranked according to its importance to the model. The higher the value is, the greater the contribution of the genus to the prediction accuracy of the model. We took the top 20 taxons of the genus to predict *via* the model and visualize by R software (**Figure 8**). The genus *Lactobacillus* was significantly higher than other genera, indicating that the intervention of *L.casei* played an important contribution in the change of intestinal flora.

### 3.5 Microbial Function Prediction

**Figure 9:**
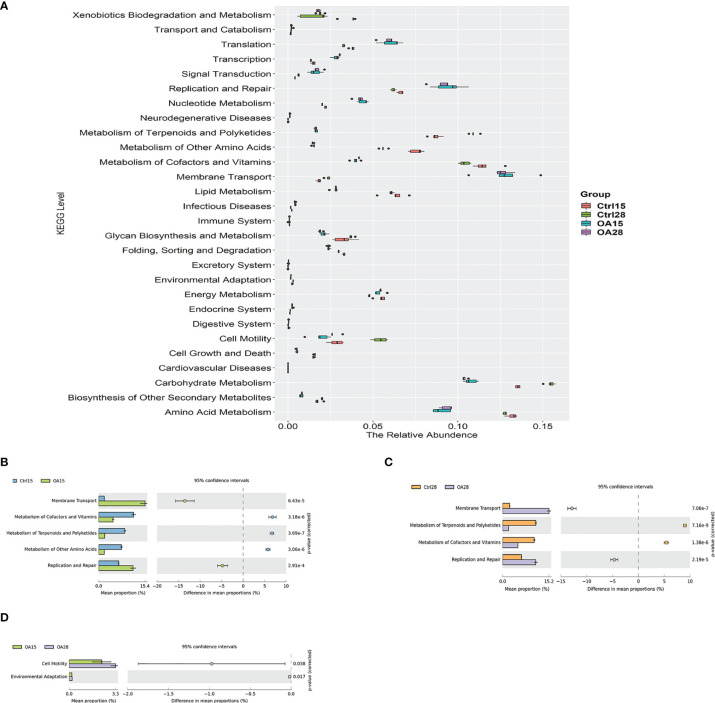
Comparisons of the predominant gene pathways of the bacterial microbiota in different groups as predicted by PICRUSt. The box diagram describes the relative abundance in each group **(A)**. The differences of metabolism among the Ctrl15 group, Ctrl28 group, and the first feeding *L.casei* groups OA15 and OA28 were analyzed by using STAMP software with two-sided Student’s t-test. Ctrl15 group vs. OA15 group **(B)**, Ctrl28 group vs. OA28 group **(C),** and OA15 group vs. OA28 group **(D)**.

16s rRNA marker gene sequences were used to predict the functional profiling of microbial communities by using Kyoto Encyclopedia of Genes and Genomes (KEGG), which were obtained through a phylogenetic prediction investigation of communities *via* reconstruction of unobserved states (PICRUSt). All the analysis results are shown in **Figure 9A**. We discovered that after the *L.casei* treatment, the relative abundances of the genes involved in membrane transport, replication, and repair were significantly increased, but in lipid metabolism, metabolism of cofactors and vitamins, metabolism of terpenoids and polyketides, and metabolism of other amino acids were significantly declined (*p* < 0.05) (**Figure 9B**). In the OA28 group, the relative abundances of the genes involved in membrane transport, replication, and repair were significantly increased (*p* < 0.05) (**Figure 9C**). In the OA15 vs. OA28 group, the relative abundances of the genes involved in cell motility and environmental adaptation were significantly decreased (**Figure 9D**).

### 3.6 Correlation Analysis Between Intestinal Microflora Composition Changes and Body Function Indexes

**Figure 10:**
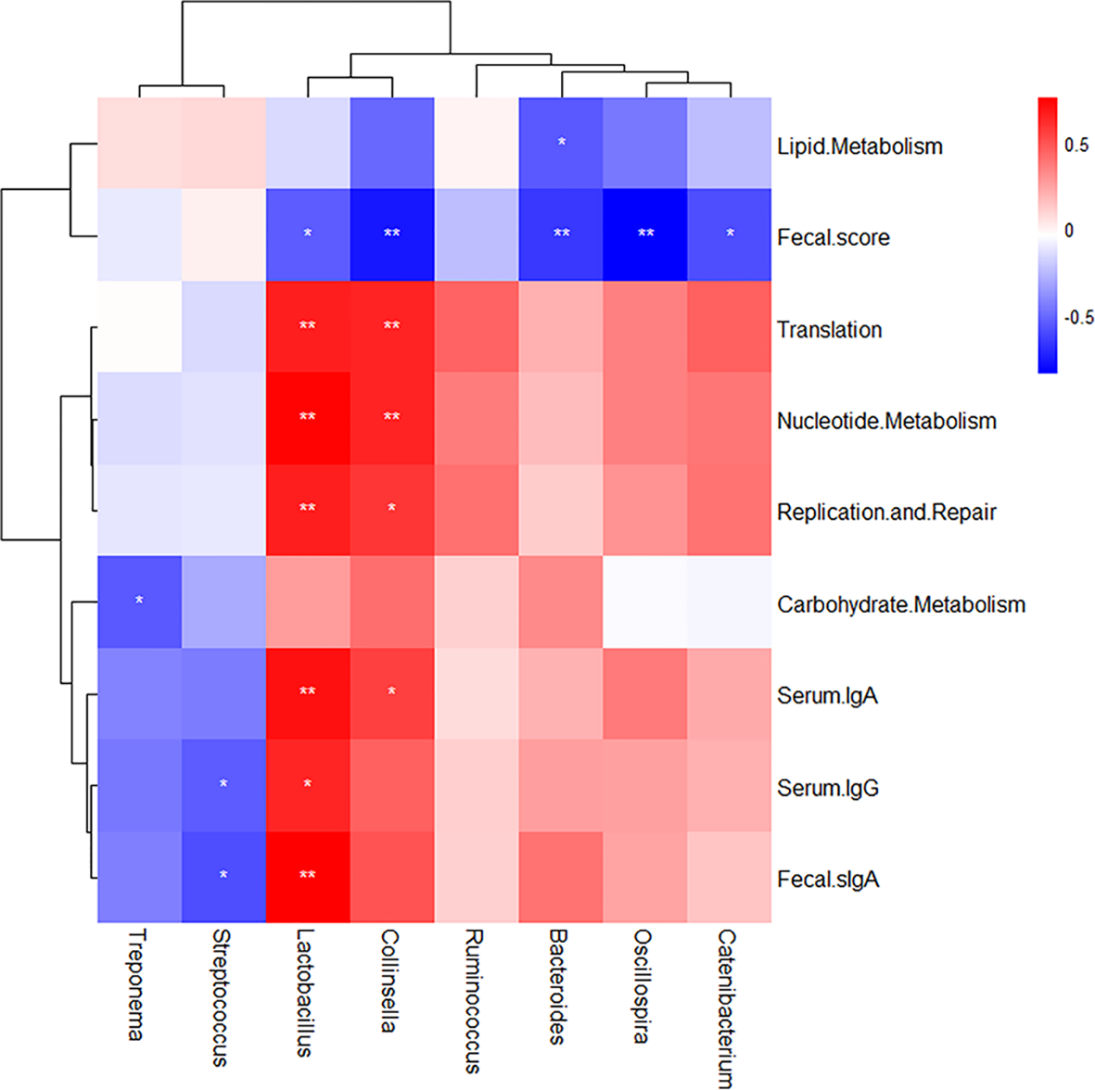
Correlation analysis between intestinal microflora composition changes and body function indexes. The Spearman rank correlation analysis was used and drawn by R software (version 3.6.1) with pheatmap package. The color legend is on the top right of the figure. Red indicates positive correlation; blue indicates negative correlation. *p < 0.05 and **p < 0.01 using Student’s t-test to evaluate differences between every two targets.

The Spearman rank correlation analysis was used to study the potential relationship between intestinal microbial composition and body function in piglets (**Figure 10**). The genus *Lactobacillus* abundance enhancement was positively correlated with the increased levels of translation, nucleotide metabolism, replication and repair, serum IgG value, serum IgA value, and fecal sIgA value (*p* < 0.05) but was negatively correlated with the fecal score (*p* < 0.05). The genus *Collinsella* abundance enhancement was also positively related to the increased levels of translation, nucleotide metabolism, replication and repair, and serum IgA value (*p* < 0.05). However, the genus *Treponema* abundance enhancement and the increased levels of carbohydrate metabolism were negatively correlated (*p* < 0.05). *Bacteroides* abundance enhancement was negatively correlated with the lipid metabolism increased level (*p* < 0.05).

## 4 Discussion

The animal body is colonized by a diverse community of microorganisms collectively referred to as the microbiota that mediate colonization resistance directly and indirectly against infectious pathogens. Probiotics have been used extensively as animal feed additives. There are several benefits set for probiotics: preventing infections (Hu et al., 2017; Lin and Zhang, 2017; Azad et al., 2018), potential antitumor activity (So et al., 2017; Yu, 2018), promoting food absorption, and repairing the intestinal epithelial barrier (Chang et al., 2018; Kim et al., 2018; Huang et al., 2019). Our studies on *L.casei*, one of the probiotics, as a live vehicle for the delivery of heterologous antigens to the mucosa have been conducted for over 20 years. In the present work, we investigated the changes of intestinal flora, growth of piglets, specific humoral immunity, and mucosal immunity under the intervention of recombinant pLA-K88/*L.casei*, which provided important insights into the diversity and function of piglets gut microbiota. We tried to find out about the therapeutic modulation of the gut microbiota in piglets to prevent or treat diarrhea diseases by recombinant *L.casei*.

In this study, we found that pLA-ETEC K88/*L.casei* were sufficient to elicit elevated sIgA responses in mucosal tissues as well as the systemic IgG antibody responses. The treatment of recombinant *L.casei* to newborn piglets induced the anti-K88-specific serum IgG, serum IgA, and fecal sIgA, suggesting that they could be the potential mucosal vaccine against ETEC infection. However, its molecular mechanism still needs to be confirmed experimentally, such as what T cell responses caused by the mucosal vaccine play an important role.

In order to evaluate the diversity of the bacterial community in newborn piglets, a series of α-diversity indexes were calculated. We found out that the richness and diversity of intestinal flora in the r*L.casei*-treated group were significantly increased on day 28. Similar results were also reported in some studies demonstrating that *L.casei* can increase the α-diversity index of the microbial ecosystem in mice (Liew et al., 2019). However, there are a very limited number of studies that have analyzed the α-diversity index of the microbial ecosystem in piglets under the intervention of recombinant *L.casei*. In β-diversity, microbiota structure (β-diversity indices) was changed after pLA-K88/*L.casei* treatment. The OA groups have formed distinct clusters, and these groups tended to separate from the Ctrl15 group. One possible explanation for this discrepancy is that it was caused by the intervention effects of pLA-K88/*L.casei*. Another possibility is that the *Lactobacillus* peptides secreted by probiotics regulated the change of intestinal flora (Hu et al., 2018; Wang et al., 2020).

More generally, our results indicate that the oral administration of pLA-K88/*L.casei* can decrease the relative abundance phyla of *Proteobacteria*. Thus, we speculate that the intervention of recombinant *L.casei* to piglets may relieve diarrhea symptoms caused by ETEC, which was proved by the decreased diarrhea rate. In previous research in our lab, pLA-K88/*L.casei* can protect mice against ETEC-K88 challenge, and 85% protective efficiency was obtained to provide a first line of protection at infectious pathogen entry ports (Wen et al., 2012). However, we still need corresponding experiments to prove the immune protection rate in piglets. Furthermore, our results also showed that the oral administration of recombinant *L.casei* not only significantly increased the relative abundance of *L.casei* but also altered the intestinal microbiota in the feces of newborn piglets, as evidenced by altered microbial diversity, microbial taxonomic composition, and bacterial functional profiles. Surprisingly, either family or genus, the richness of *Lactobacillus* was decreased in the OA28 group. One possible reason is that decreasing antimicrobial peptides owing to the low-abundance *Lactobacilli* caused by the oral tolerance mechanism further resulted in changes in relative abundance of the dominant genus. It is generally believed that the gut immune system normally does not respond to food antigens and the gut’s native bacteria, which is called oral tolerance. This tolerance state is closely associated with various components of intestinal epithelial cell integrity, dendritic cell presentation, CD4+CD25+Treg cells, and other factors, which may influence the balance of the microenvironment in gut. Furthermore, previous studies reported that probiotics stimulated Paneth cells to produce antimicrobial peptides and regulated the relative stability of intestinal flora (Chen et al., 2017; Lueschow et al., 2018; Wang et al., 2018a). On all accounts, solving this problem needs our further experimental studies. Also, further research is required to clarify the molecular basis of the microbiota variation in a larger scale of piglets.

Our results demonstrated that the OA15 cluster was close to the Ctrl28 cluster (**Figure 5**). The possible reason was that supplemented probiotics were part of the “transient microbiota” in the pigs’ body for a relatively short period, which was consistent with our previous findings. The permanent colonization of the supplemented probiotics is largely hindered by the resident flora, suggesting that continuous addition of the probiotics might be an option for farm applications. Interestingly, our data suggested that after administration of pLA-K88/*L.casei*, the functional alteration of the intestinal microbiota was characterized by significantly increasing and positive correlating with membrane transport, repair, and translation. This indicated that the *L.casei* intervention to piglets could enhance the host transport of intracellular substances and cell proliferation and repair, which can restore the intestinal mucosal barrier, relieve diarrhea, and enhance immune function to an improved clinical outcome of diarrheal disease (Wang et al., 2017; Riaz Rajoka et al., 2018; Wang et al., 2019).

Thus, oral administration of pLA-K88/*L.casei* facilitates the functional maturation of the intestinal microbiota in newborn piglets. To sum up, this study revealed that the recombinant *Lactobacillus casei* was worthwhile to promote the shape of intestinal flora in newborn piglets, although further experimental studies are still needed to uncover the recombinant *L.casei* impact on host microbiome interaction and on the molecular mechanism and cellular pathway of repairing the host intestinal mucosal barrier. This beneficial superimposed effect of the specific immunity and probiotic role will be a strategy for preventing piglets from diarrhea.

## Data Availability Statement

The data presented in the study are deposited in the NCBI Sequence Read Archive (SRA) database, accession number SRP282258 and SRP344853.

## Ethics Statement

The animal study was reviewed and approved by the Animal Care and Ethics Committee of Heilongjiang Bayi Agricultural University (HBAU-2019003).

## Author Contributions

L-YY, XH, GW, and LL contributed to the conception and design of the study. DQ, YB, LY, and YH conducted the experiments and analyzed the experimental results. YQ and JW performed the statistical analysis. DQ, YB, and L-YY wrote the first draft of the manuscript. All of the authors contributed to the manuscript revision and read and approved the submitted version.

## Funding

This study was supported by the Scientific Research Foundation for the Heilongjiang Natural Science Foundation (Grant number C2017047) and the scientific research team support plan of Heilongjiang Bayi Agricultural University (TDJH201904).

## Conflict of Interest

The authors declare that the research was conducted in the absence of any commercial or financial relationships that could be construed as a potential conflict of interest.

## Publisher’s Note

All claims expressed in this article are solely those of the authors and do not necessarily represent those of their affiliated organizations, or those of the publisher, the editors and the reviewers. Any product that may be evaluated in this article, or claim that may be made by its manufacturer, is not guaranteed or endorsed by the publisher.

## References

[B1] Azad M. A. K. Sarker M. Wan D. (2018). Immunomodulatory Effects of Probiotics on Cytokine Profiles. BioMed. Res. Int. 2018, 8063647. doi: 10.1155/2018/8063647 30426014 PMC6218795

[B2] Bai Y. Wang G. Qi H. Wang Y. Xu C. Yue L.. (2020). Immunogenicity of 987P Fimbriae of Enterotoxigenic Escherichia Coli Surface-Displayed on Lactobacillus Casei. Res. Vet. Sci. 128, 308–314. doi: 10.1016/j.rvsc.2019.12.016 31901569

[B3] Bokulich N. A. Subramanian S. Faith J. J. Gevers D. Gordon J. I. Knight R.. (2013). Quality-Filtering Vastly Improves Diversity Estimates From Illumina Amplicon Sequencing. Nat. Methods 10, 57–59. doi: 10.1038/nmeth.2276 23202435 PMC3531572

[B4] Breiman L. (2001). Random Forests. Mach. Learn 45, 5–32. doi: 10.1023/A:1010933404324

[B5] Caporaso J. G. Kuczynski J. Stombaugh J. Bittinger K. Bushman F. D. Costello E. K.. (2010). QIIME Allows Analysis of High-Throughput Community Sequencing Data. Nat. Methods 7, 335–336. doi: 10.1038/nmeth.f.303 20383131 PMC3156573

[B6] Chang C. W. Liu C. Y. Lee H. C. Huang Y. H. Li L. H. Chiau J. S. C.. (2018). *Lactobacillus Casei* Variety Rhamnosus Probiotic Preventively Attenuates 5-Fluorouracil/Oxaliplatin-Induced Intestinal Injury in a Syngeneic Colorectal Cancer Model. Front. Microbiol. 9. doi: 10.3389/fmicb.2018.00983 PMC5962742 29867884

[B7] Chen J. Huang C. Wang J. Zhou H. Lu Y. Lou L.. (2017). Dysbiosis of Intestinal Microbiota and Decrease in Paneth Cell Antimicrobial Peptide Level During Acute Necrotizing Pancreatitis in Rats. PloS One 12, e0176583. doi: 10.1371/journal.pone.0176583 28441432 PMC5404871

[B8] Chen X. Xu J. Ren E. Su Y. Zhu W. (2018). Co-Occurrence of Early Gut Colonization in Neonatal Piglets With Microbiota in the Maternal and Surrounding Delivery Environments. Anaerobe 49, 30–40. doi: 10.1016/j.anaerobe.2017.12.002 29223548

[B9] De Filippo C. Cavalieri D. Di Paola M. Ramazzotti M. Poullet J. B. Massart S.. (2010). Impact of Diet in Shaping Gut Microbiota Revealed by a Comparative Study in Children From Europe and Rural Africa. Proc. Natl. Acad. Sci. U.S.A. 107, 14691–14696. doi: 10.1073/pnas.1005963107 20679230 PMC2930426

[B10] Dorsey F. Fischer J. Fleckenstein J. (2010). Directed Delivery of Heat-Labile Enterotoxin by Enterotoxigenic *Escherichia Coli* . Cell Microbiol. 8, 1516–1527. doi: 10.1111/j.1462-5822.2006.00736.x 16922869

[B11] Edgar R. C. (2010). Search and Clustering Orders of Magnitude Faster Than BLAST. Bioinformatics 26, 2460–2461. doi: 10.1093/bioinformatics/btq461 20709691

[B12] Friedman R. S. Frankel F. R. Xu Z. Lieberman J. (2000). Induction of Human Immunodeficiency Virus (HIV)-Specific CD8 T-Cell Responses by *Listeria* Monocytogenes and a Hyperattenuated *Listeria* Strain Engineered to Express HIV Antigens. J. Virol. 74, 9987–9993. doi: 10.1128/JVI.74.21.9987-9993.2000 11024127 PMC102037

[B13] Geng H. Shu S. Dong J. Li H. Xu C. Han Y.. (2018). Association Study of Gut Flora in Wilson’s Disease Through High-Throughput Sequencing. Med. (United States) 97, e11743. doi: 10.1097/MD.0000000000011743 PMC6081054 30075590

[B14] Gresse R. Chaucheyras-Durand F. Fleury M. A. Van de Wiele T. Forano E. Blanquet-Diot S. (2017). Gut Microbiota Dysbiosis in Postweaning Piglets: Understanding the Keys to Health. Trends Microbiol. 25, 851–873. doi: 10.1016/j.tim.2017.05.004 28602521

[B15] Guevarra R. B. Lee J. H. Lee S. H. Seok M. J. Kim D. W. Kang B. N.. (2019). Piglet Gut Microbial Shifts Early in Life: Causes and Effects. J. Anim. Sci. Biotechnol. 10, 1. doi: 10.1186/s40104-018-0308-3 30651985 PMC6330741

[B16] Ho P. S. Kwang J. Lee Y. K. (2005). Intragastric Administration of *Lactobacillus Casei* Expressing Transmissible Gastroentritis Coronavirus Spike Glycoprotein Induced Specific Antibody Production. Vaccine 23, 1335–1342. doi: 10.1016/j.vaccine.2004.09.015 15661381 PMC7115493

[B17] Huang L. Chiang Chiau J. S. Cheng M. L. Chan W. T. Jiang C. B. SW C.. (2019). SCID/NOD Mice Model for 5-FU Induced Intestinal Mucositis: Safety and Effects of Probiotics as Therapy. Pediatr. Neonatol. 60, 252–260. doi: 10.1016/j.pedneo.2018.07.007 30150027

[B18] Hu J. Ma L. Nie Y. Chen J. Zheng W. Wang X.. (2018). A Microbiota-Derived Bacteriocin Targets the Host to Confer Diarrhea Resistance in Early-Weaned Piglets. Cell Host Microbe 24, 817–832.e8. doi: 10.1016/j.chom.2018.11.006 30543777

[B19] Hu S. Wang L. Jiang Z. (2017). Dietary Additive Probiotics Modulation of the Intestinal Microbiota. Protein Pept. Lett. 24, 382–387. doi: 10.2174/0929866524666170223143615 28240160

[B20] Ikwap K. Larsson J. Jacobson M. Owiny D. O. Nasinyama G. W. Nabukenya I.. (2016). Prevalence of Adhesin and Toxin Genes in E. Coli Strains Isolated From Diarrheic and Non-Diarrheic Pigs From Smallholder Herds in Northern and Eastern Uganda. BMC Microbiol. 16, 1–9. doi: 10.1186/s12866-016-0796-2 27496201 PMC4974785

[B21] Kim N. Yun M. Oh Y. J. Choi H. J. (2018). Mind-Altering With the Gut: Modulation of the Gut-Brain Axis With Probiotics. J. Microbiol. 56, 172–182. doi: 10.1007/s12275-018-8032-4 29492874

[B22] Kuczkowska K. Myrbråten I. Øverland L. Eijsink V. G. H. Follmann F. Mathiesen G.. (2017). *Lactobacillus Plantarum* Producing a *Chlamydia Trachomatis* Antigen Induces a Specific IgA Response After Mucosal Booster Immunization. PloS One 12, 1–16. doi: 10.1371/journal.pone.0176401 PMC5415134 28467432

[B23] Langille M. G. I. Zaneveld J. Caporaso J. G. McDonald D. Knights D. Reyes J. A.. (2013). Predictive Functional Profiling of Microbial Communities Using 16S rRNA Marker Gene Sequences. Nat. Biotechnol. 31, 814–821. doi: 10.1038/nbt.2676 23975157 PMC3819121

[B24] LeCureux J. S. Dean G. A. (2018). *Lactobacillus* Mucosal Vaccine Vectors: Immune Responses Against Bacterial and Viral Antigens. mSphere 3, 1–15. doi: 10.1128/msphere.00061-18 PMC5956152 29769376

[B25] Lee J. S. Shin K. S. Pan J. G. Kim C. J. (2000). Surface-Displayed Viral Antigens on *Salmonella* Carrier Vaccine. Nat. Biotechnol. 18, 645. doi: 10.1038/76494 10835603

[B26] Liew W. P. P. Mohd-Redzwan S. Than L. T. L. (2019). Gut Microbiota Profiling of Aflatoxin B1-Induced Rats Treated With *Lactobacillus Casei* Shirota. Toxins (Basel) 11, 49. doi: 10.3390/toxins11010049 PMC6357033 30658400

[B27] Li Y. Guo Y. Wen Z. Jiang X. Ma X. Han X. (2018). Weaning Stress Perturbs Gut Microbiome and Its Metabolic Profile in Piglets. Sci. Rep. 8, 18068. doi: 10.1038/s41598-018-33649-8 30584255 PMC6305375

[B28] Lin L. Zhang J. (2017). Role of Intestinal Microbiota and Metabolites on Gut Homeostasis and Human Diseases. BMC Immunol. 18, 2. doi: 10.1186/s12865-016-0187-3 28061847 PMC5219689

[B29] Lozupone C. Knight R. (2005). UniFrac: A New Phylogenetic Method for Comparing Microbial Communities. Appl. Environ. Microbiol. 71, 8228–8235. doi: 10.1128/AEM.71.12.8228-8235.2005 16332807 PMC1317376

[B30] Lueschow S. R. Stumphy J. Gong H. Kern S. L. Elgin T. G. Underwood M. A.. (2018). Loss of Murine Paneth Cell Function Alters the Immature Intestinal Microbiome and Mimics Changes Seen in Neonatal Necrotizing Enterocolitis. PloS One 13, e0204967. doi: 10.1371/journal.pone.0204967 30273395 PMC6166990

[B31] Lundberg D. S. Yourstone S. Mieczkowski P. Jones C. D. Dangl J. L. (2013). Practical Innovations for High-Throughput Amplicon Sequencing. Nat. Methods 10, 999–1002. doi: 10.1038/nmeth.2634 23995388

[B32] McFarland L. V. Ship N. Auclair J. Millette M. (2018). Primary Prevention of *Clostridium Difficile* Infections With a Specific Probiotic Combining *Lactobacillus Acidophilus*, L. Casei, and *L. Rhamnosus* Strains: Assessing the Evidence. J. Hosp Infect. 99, 443–452. doi: 10.1016/j.jhin.2018.04.017 29702133

[B33] Moon H. W. Isaacson R. E. Pohlenz J. (1979). Mechanisms of Association of Enteropathogenic *Escherichia Coli* With Intestinal Epithelium. Am. J. Clin. Nutr. 32, 119. doi: 10.1093/ajcn/32.1.119 367139

[B34] Moon H. W. Nagy B. Isaacson R. E. Orskov I. (1977). Occurrence of K99 Antigen on *Escherichia Coli* Isolated From Pigs and Colonization of Pig Ileum by K99+ Enterotoxigenic E. Coli From Calves and Pigs. Infect. Immun. 15, 614–620. doi: 10.1128/iai.15.2.614-620.1977 321356 PMC421411

[B35] Morris J. A. Thorns C. J. Wells G. A. Scott A. C. Sojka W. J. (1983). The Production of F41 Fimbriae by Piglet Strains of Enterotoxigenic *Escherichia Coli* That Lack K88, K99 and 987P Fimbriae. J. Gen. Microbiol. 129, 2753. doi: 10.1099/00221287-129-9-2753 6138394

[B36] Ou D. Li D. Cao Y. Li X. Yin J. Qiao S.. (2007). Dietary Supplementation With Zinc Oxide Decreases Expression of the Stem Cell Factor in the Small Intestine of Weanling Pigs. J. Nutr. Biochem. 18, 820–826. doi: 10.1016/j.jnutbio.2006.12.022 17475461

[B37] Pouwels P. H. Leer R. J. Boersma W. J. A. (1996). The Potential of *Lactobacillus* as a Carrier for Oral Immunization: Development and Preliminary Characterization of Vector Systems for Targeted Delivery of Antigens. J. Biotechnol. 44, 183–192. doi: 10.1016/0168-1656(95)00140-9 8717402

[B38] Pouwels P. H. Leer R. J. Shaw M. Heijne Den Bak-Glashouwer M. J. Tielen F. D. Smit E.. (1998). Lactic Acid Bacteria as Antigen Delivery Vehicles for Oral Immunization Purposes. Int. J. Food Microbiol. 41, 155–167. doi: 10.1016/S0168-1605(98)00048-8 9704864

[B39] Praveen M. Jones K. F. Geller B. L. (2004). Mucosal Vaccine Made From Live, Recombinant *Lactococcus Lactis* Protects Mice Against Pharyngeal Infection With Streptococcus Pyogenes. Infect. Immun. 72, 3444. doi: 10.1128/IAI.72.6.3444-3450.2004 15155651 PMC415684

[B40] Quast C. Pruesse E. Yilmaz P. Gerken J. Schweer T. Yarza P.. (2013). The SILVA Ribosomal RNA Gene Database Project: Improved Data Processing and Web-Based Tools. Nucleic Acids Res. 41, D590–D596. doi: 10.1093/nar/gks1219 23193283 PMC3531112

[B41] Ramette A. (2007). Multivariate Analyses in Microbial Ecology. FEMS Microbiol. Ecol. 62, 142–160. doi: 10.1111/j.1574-6941.2007.00375.x 17892477 PMC2121141

[B42] Riaz Rajoka M. S. Zhao H. Lu Y. Lian Z. Li N. Hussain N.. (2018). Anticancer Potential Against Cervix Cancer (HeLa) Cell Line of Probiotic: *Lactobacillus Casei* and *Lactobacillus Paracasei* Strains Isolated From Human Breast Milk. Food Funct. 9, 2705–2715. doi: 10.1039/c8fo00547h 29762617

[B43] Sahagun-Ruiz A. Velazquez L. V. Bhaskaran S. Jay C. M. Morales-Salinas E. Rathore K.. (2015). Reduction of Enterotoxin Induced Fluid Accumulation in Ileal Loops of Neonatal Calves With Anti-F5 Fimbriae Recombinant Antibody. Vet. Res. Commun. 39, 229–236. doi: 10.1007/s11259-015-9646-1 26521056

[B44] Seegers J. F. M. L. (2002). *Lactobacilli* as Live Vaccine Delivery Vectors: Progress and Prospects. Trends Biotechnol. 20, 508–515. doi: 10.1016/S0167-7799(02)02075-9 12443872

[B45] Shata M. T. Hone D. M. (2001). Vaccination With a Shigella DNA Vaccine Vector Induces Antigen-Specific CD8+ T Cells and Antiviral Protective Immunity. J. Virol. 75, 9665–9670. doi: 10.1128/JVI.75.20.9665-9670.2001 11559798 PMC114537

[B46] So S. S. Y. Wan M. L. Y. El-Nezami H. (2017). Probiotics-Mediated Suppression of Cancer. Curr. Opin. Oncol. 29, 62–72. doi: 10.1097/CCO.0000000000000342 27792053

[B47] Tarahomjoo S. (2012). Development of Vaccine Delivery Vehicles Based on Lactic Acid Bacteria. Mol. Biotechnol. 51, 183–199. doi: 10.1007/s12033-011-9450-2 21901278 PMC7090618

[B48] Wang M. Gao Z. Zhang Y. Pan L. (2016). Lactic Acid Bacteria as Mucosal Delivery Vehicles: A Realistic Therapeutic Option. Appl. Microbiol. Biotechnol. 100, 5691–5701. doi: 10.1007/s00253-016-7557-x 27154346

[B49] Wang G. Li X. Zhao J. Zhang H. Chen W. (2017). *Lactobacillus Casei* CCFM419 Attenuates Type 2 Diabetes *via* a Gut Microbiota Dependent Mechanism. Food Funct. 8, 3155–3164. doi: 10.1039/c7fo00593h 28782784

[B50] Wang J. Tian F. Wang P. Zheng H. Zhang Y. Tian H.. (2018a). Gut Microbiota as a Modulator of Paneth Cells During Parenteral Nutrition in Mice. J. Parenter Enter Nutr. 42, 1280–1287. doi: 10.1002/jpen.1162 29701912

[B51] Wang Y. Xia S. T. Tang Q. Wu J. Zhu X. (2018b). A Novel Consistent Random Forest Framework: Bernoulli Random Forests. IEEE Trans. Neural Networks Learn Syst. 29, 3510–3523. doi: 10.1109/TNNLS.2017.2729778 28816676

[B52] Wang Y. Yan X. Zhang W. Liu Y. Han D. Teng K.. (2019). *Lactobacillus Casei* Zhang Prevents Jejunal Epithelial Damage to Early-Weaned Piglets Induced by *Escherichia Coli* K88 *via* Regulation of Intestinal Mucosal Integrity, Tight Junction Proteins and Immune Factor Expression. J. Microbiol. Biotechnol. 29, 863–876. doi: 10.4014/jmb.1903.03054 31091863

[B53] Wang G. Yu Y. Garcia-gutierrez E. Jin X. He Y. Wang L.. (2020). *Lactobacillus Acidophilus* JCM 1132 Strain and Its Mutant With Different Bacteriocin-Producing Behaviour Have Various *In Situ* Effects on the Gut Microbiota of Healthy Mice. Microorganisms 8, 49. doi: 10.3390/microorganisms8010049 PMC7022661 31881756

[B54] Wen L. J. Hou X. L. Wang G. H. Yu L. Y. Wei X. M. Liu J. K.. (2012). Immunization With Recombinant Lactobacillus Casei Strains Producing K99, K88 Fimbrial Protein Protects Mice Against Enterotoxigenic *Escherichia Coli* . Vaccine 30, 3339–3349. doi: 10.1016/j.vaccine.2011.08.036 21856357

[B55] Wu G. D. Chen J. Hoffmann C. Bittinger K. Chen Y. Y. Keilbaugh S. A.. (2011). Linking Long-Term Dietary Patterns With Gut Microbial Enterotypes. Science 80-) 334, 105–108. doi: 10.1126/science.1208344 PMC3368382 21885731

[B56] Yu L. C. H. (2018). Microbiota Dysbiosis and Barrier Dysfunction in Inflammatory Bowel Disease and Colorectal Cancers: Exploring a Common Ground Hypothesis. J. BioMed. Sci. 25, 79. doi: 10.1186/s12929-018-0483-8 30413188 PMC6234774

[B57] Zhang J. Zhang N. Liu Y. X. Zhang X. Hu B. Qin Y.. (2018). Root Microbiota Shift in Rice Correlates With Resident Time in the Field and Developmental Stage. Sci. China Life Sci. 61, 613–621. doi: 10.1007/s11427-018-9284-4 29582350

